# Supplementary figures and images for: Genomic and Phenotypic Characterization of Cutibacterium acnes Bacteriophages Isolated from Acne Patients
Source: Antibiotics (Basel). 2022 Aug 2;11(8):1041. doi: 10.3390/antibiotics11081041 (PMC9404880; doi:10.3390/antibiotics11081041)

Supplementary Figure S1.

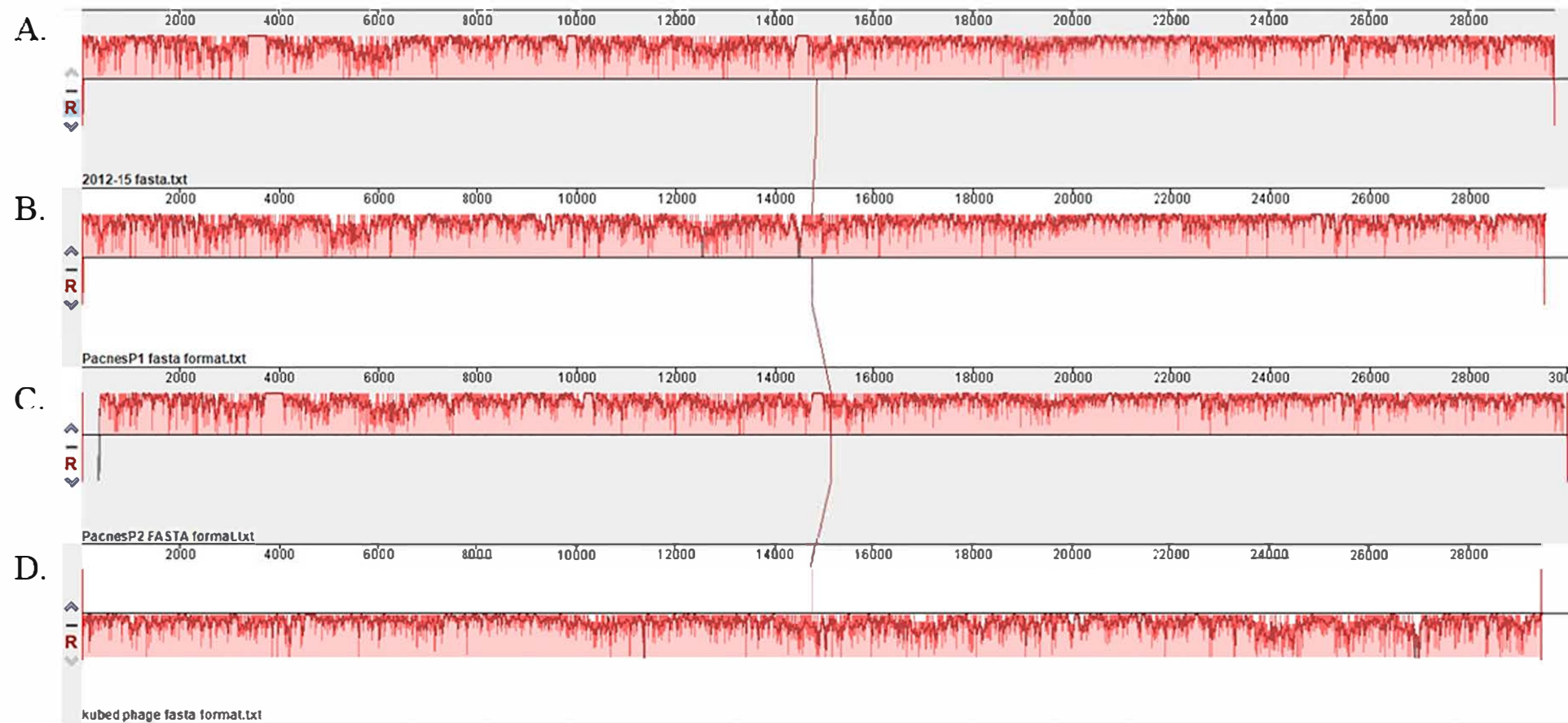

Supplement: Supplementary file 1 [file antibiotics-11-01041-s001.zip › suppl Figure S1.pdf]

Supplementary Figure S2.

A.

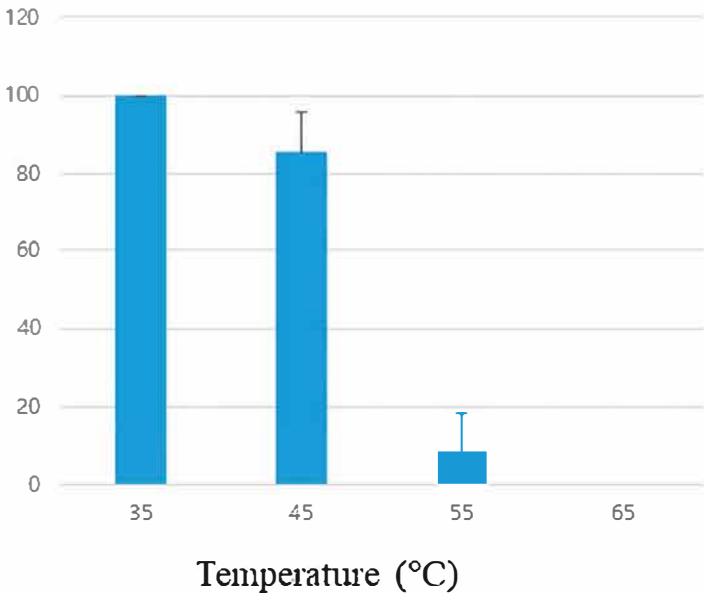

B.

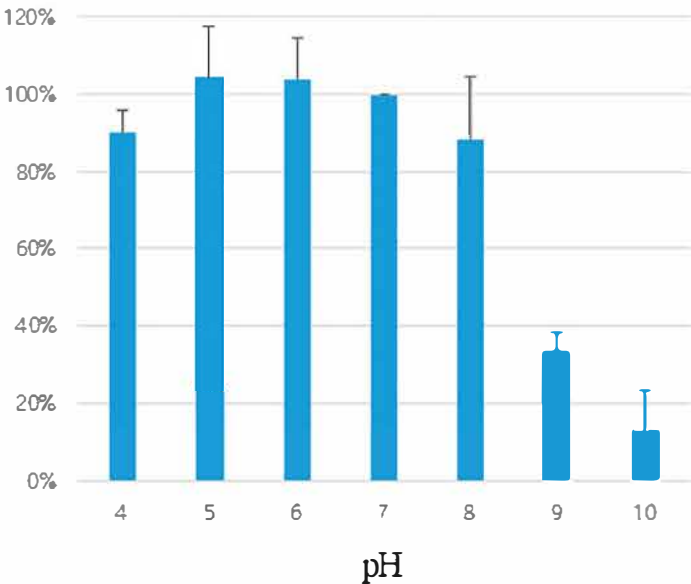

Supplement: Supplementary file 1 [file antibiotics-11-01041-s001.zip › suppl Figure S2.pdf]
